# Cardiopulmonary Complications in Obese Patients with Gynecologic Cancer: A Narrative Review

**DOI:** 10.3390/life16020323

**Published:** 2026-02-13

**Authors:** Maria Fanaki, Nikolaos Thomakos, Vasileios Lygizos, Antonia Varthaliti, Dimitrios Haidopoulos, Dimitrios Efthimios Vlachos, Vasileios Pergialiotis

**Affiliations:** First Department of Obstetrics and Gynecology, Division of Gynecologic Oncology, “Alexandra” General Hospital, National and Kapodistrian University of Athens, 115 28 Athens, Greece; thomakir@hotmail.com (N.T.); vlygizos@yahoo.com (V.L.); antonia.varthaliti@hotmail.com (A.V.); dimitrioshaidopoulos@gmail.com (D.H.); vlachosdg@gmail.com (D.E.V.)

**Keywords:** obesity, gynecologic cancer patients, cardiovascular disease, pulmonary complications, cardiopulmonary

## Abstract

Gynecologic cancer is a major global health burden, and improvements in screening, surgical techniques, and systemic therapies have significantly prolonged survival. As a result, cardiopulmonary disease has emerged as a leading non-cancer cause of morbidity and mortality among gynecologic cancer survivors. Obesity, which is highly prevalent in this population, substantially increases cardiopulmonary risk by contributing to metabolic syndrome, cardiovascular disease, chronic inflammation, and reduced cardiopulmonary reserve. This narrative review summarizes current evidence on the epidemiology, pathophysiological mechanisms, and clinical spectrum of cardiopulmonary complications in obese patients with gynecologic malignancy. We review the contribution of obesity-related metabolic and endothelial dysfunction, cancer-associated hypercoagulability, and treatment-related toxicities, with particular emphasis on complications arising from surgery, chemotherapy, and radiotherapy. Epidemiologic data demonstrate a markedly increased burden of cardiovascular and pulmonary disease in obese gynecologic cancer patients, including higher rates of myocardial injury, heart failure, venous thromboembolism, and postoperative respiratory complications. Surgical treatment, although central to oncologic management, imposes substantial cardiopulmonary stress, placing obese patients at heightened perioperative risk. Future studies should focus on preoperative risk stratification, optimization of obesity-related comorbidities, and multidisciplinary perioperative management, including enhanced recovery pathways, as well as appropriate use of high-dependency or intensive care monitoring to reduce morbidity and improve both oncologic and long-term non-oncologic outcomes in this population.

## 1. Introduction

Gynecologic cancer is a significant global health issue accounting for approximately 16% of all cancers in women [1]. Based on GLOBOCAN 2022, cervical cancer is the most common gynecologic malignancy, with about 662,000 new cases annually, followed by endometrial (uterine body) cancer with 420,000 cases and ovarian cancer with 325,000 cases per year. Improvements in screening, surgical techniques, systemic treatment, and radiotherapy have significantly enhanced survival outcomes, resulting in a growing emphasis on survivorship and long-term health issues. Consequently, cardiovascular and pulmonary disease has become one of the most important non-cancer causes of morbidity and mortality among cancer survivors [2,3,4].

Population-based studies have shown that cancer survivors face up to a twofold increased risk of cardiovascular disease (CVD)-related mortality, especially within the first year after diagnosis, with this elevated risk persisting in the long term [2]. Gynecological cancer patients are particularly vulnerable to cardiopulmonary complications due to the cardiotoxic effects of chemotherapy and radiotherapy, as well as shared risk factors such as obesity, smoking, hypertension, and aging [5,6]. In particular, survivors of gynecologic cancer appear to have an elevated risk of CVD mortality compared with the general population, and in patients with endometrial or ovarian cancer, cardiovascular death may exceed cancer-related mortality over time [3,4,7].

In parallel, obesity substantially increases the risk of gynecologic cancer by promoting a pro-carcinogenic environment characterized by unopposed estrogen exposure, hyperinsulinemia, and chronic systemic inflammation [8], while also representing a major underlying contributor to metabolic syndrome and cardiovascular disease. Population-based studies demonstrate that cancer survivors experience up to a twofold increased risk of cardiovascular disease–related mortality compared with the general population, particularly within the first year following diagnosis, with this elevated risk persisting long term. Gynecologic cancer survivors appear especially vulnerable due to shared cardiometabolic risk factors and exposure to potentially cardiotoxic treatments, and in selected malignancies such as endometrial and ovarian cancer, cardiovascular mortality may exceed cancer-related mortality over time [5,9].

The aim of this narrative review is to synthesize current evidence on the epidemiology, underlying mechanisms, and clinical spectrum of cardiopulmonary complications in obese patients with gynecologic malignancies, with the goal of enhancing clinical awareness, supporting risk stratification, and informing multidisciplinary strategies to reduce cardiopulmonary morbidity and improve long-term outcomes in this population.

## 2. Methods and Materials

This narrative review aims to investigate current evidence on cardiopulmonary complications of obese gynecological cancer patients. A comprehensive literature search was conducted using electronic databases including PubMed, Scopus, and Google Scholar. The search was limited to English-language publications published between September 1986 and July 2025. Keywords and MeSH terms included “obesity,” “gynecologic cancer patients,” “cardiovascular disease,” “pulmonary complications,” and “cardiopulmonary” with Boolean operators (AND, OR) applied to refine the search strategy.

Eligible studies included original research articles, systematic reviews, meta-analyses, and clinical guidelines addressing the epidemiology, pathophysiology, clinical implications, assessment, and management of cardiopulmonary complications in obese gynecologic cancer patients. Case reports, editorials, letters to the editor, studies limited to pediatric populations, and articles focusing exclusively on non-surgical scenarios were excluded.

The literature search yielded approximately 280 articles after removal of duplicates. Following title and abstract screening, 224 articles underwent full-text review, of which 56 were deemed relevant and included in this narrative review. Greater emphasis was placed on high-quality systematic reviews, large cohort studies, and clinical guidelines published from 2022 onward to ensure contemporary relevance.

Titles and abstracts of retrieved studies were screened for relevance, followed by full-text review to determine eligibility. Study selection was performed independently by two reviewers (MF and VP), with disagreements resolved through consensus or consultation with a third reviewer. Data were extracted on the epidemiology of cardiovascular and pulmonary disease in gynecologic cancer patients, clinical outcomes, and the pathophysiological mechanisms linking obesity, gynecologic cancer, and cardiopulmonary complications.

## 3. Epidemiology

Cardiovascular disease (CVD) is the leading cause of morbidity and mortality among individuals with obesity, accounting for over two-thirds of the ~4.0 million deaths annually attributable to high body mass index worldwide. Obesity independently increases the risk of coronary artery disease, heart failure, stroke, atrial fibrillation, and sudden cardiac death. Epidemiologic data show that each 5 kg/m^2^ increase in BMI raises the risk of heart failure by 5–7%, sudden cardiac death by 16%, and atrial fibrillation by 29% [10]. Obesity contributes to millions of premature deaths annually, and epidemiologic studies consistently show that higher body mass index (BMI) is linked to increased cancer-specific and overall mortality in women with gynecologic malignancies. The current literature has demonstrated that obese gynecological cancer patients present worse overall survival, with the strongest and most consistent effects seen in endometrial cancer, where obesity increases mortality risk by 30–60%**,** particularly in women with morbid obesity (BMI ≥40 kg/m^2^). In cervical cancer, obese and morbidly obese patients report up to a twofold higher risk of death compared with normal-weight women. In ovarian cancer, obesity exhibited an approximately 17% higher risk of cancer-related mortality, especially in advanced-stage disease, and is associated with poorer progression-free and overall survival [11].

The results of a surveillance system survey showed that gynecologic cancer survivors demonstrate a markedly higher prevalence of obesity (46.0%) compared with women without cancer (32.7%) and survivors of other cancers (32.3%). Cardiovascular morbidity was significantly elevated among gynecologic cancer survivors, who demonstrated a 2.7-fold higher likelihood of myocardial infarction, a 3.4-fold increased risk of coronary heart disease or angina, and a 2.7-fold greater prevalence of stroke compared with women without a history of cancer [12].

In particular, cardiovascular disease (CVD) is a significant non-cancer cause of death among ovarian cancer patients. In a large SEER database analysis of approximately 110,000 women diagnosed between 2000 and 2019, cardiovascular causes accounted for 3003 deaths (5.48%)**,** compared with 54,829 cancer-related deaths. Cardiovascular mortality was more common in patients with advanced disease, particularly those with distant metastases (45.8%) and poorly differentiated tumors (24.5%). Multivariable analysis identified age >60 years as a major risk factor, while patients aged 31–60 had substantially lower risk. Additional independent risk factors included delayed diagnosis, non-white race, bilateral ovarian involvement, and regional or distant-stage disease, with metastatic disease conferring more than a twofold increase in risk of cardiovascular death [3].

The findings for endometrial cancer patients were concordant with prior evidence. A population cohort study of 157,496 endometrial cancer patients diagnosed between 1988 and 2012 showed that CVD accounted for 20.5% of all deaths, compared with 40.6% from endometrial cancer and 18.7% from other cancers. Overall, women with endometrial cancer were 8.8 times more likely to die from CVD than women in the general population (SMR 8.8, 95% CI 8.7–9.0). CVD mortality was independently associated with older age, Black race, lower household income, and lack of surgical treatment, while hysterectomy was associated with reduced CVD risk. Advanced stage or aggressive histology of endometrial cancer presented higher relative increase in CVD death. In addition, CVD mortality in patients with early-stage endometrioid cancer surpassed cancer mortality as the leading cause of death approximately five years after diagnosis [4].

Overall, these epidemiologic data indicate that obesity and cardiovascular disease are highly prevalent and interlinked contributors to adverse outcomes in women with gynecologic cancer and have a negative impact on overall survival and clinical outcomes of this group of patients.

## 4. Pathophysiological Mechanisms Linking Obesity to Cardiopulmonary Complications

In the oncologic setting, obesity-related cardiopulmonary vulnerability does not exist in isolation but interacts dynamically with cancer biology and treatment exposure. Chronic inflammation, metabolic dysregulation, and endothelial dysfunction associated with obesity not only increase baseline cardiovascular and pulmonary risk, but also amplify cancer-associated hypercoagulability, treatment-related toxicity, and perioperative stress. These mechanisms contribute to reduced treatment tolerance, higher rates of dose modification or discontinuation, and increased non-cancer mortality, thereby directly influencing oncologic outcomes and survivorship.

### 4.1. Chronic Inflammation and Endothelial Dysfunction

In obese gynecological cancer patients, chronic low-grade inflammation, endothelial dysfunction, and metabolic dysregulation represent central pathophysiological mechanisms linking obesity to heightened cardiopulmonary morbidity. Obesity, particularly central or visceral adiposity, is strongly associated with insulin resistance and compensatory hyperinsulinemia, characterized by increased visceral fat accumulation, adipocyte hypertrophy, macrophage infiltration of omental adipose tissue, and dysregulated secretion of free fatty acids and pro-inflammatory adipokines, including tumor necrosis factor-α, leptin, resistin, and plasminogen activator inhibitor-1 [13,14]. Metabolic dysregulation and hyperglycemia further impair innate immune function by disrupting neutrophil activity and enhancing oxidative stress through excessive generation of reactive oxygen species and inflammatory mediators, thereby promoting immune and vascular dysfunction [15,16]. Adipose tissue itself functions as a chronic inflammatory organ, releasing cytokines such as interleukin-6, tumor necrosis factor-α, and C-reactive protein, which drive systemic endothelial activation and compromise vascular homeostasis [17].

This persistent inflammatory signaling reduces nitric oxide bioavailability and increases oxidative stress, leading to endothelial stiffening and increased vascular permeability, which contribute to endothelial dysfunction, atherosclerosis, hypertension, and heightened susceptibility to perioperative myocardial injury and thrombotic events [18]. Importantly, endothelial dysfunction is also closely linked to impaired pulmonary function, even in the absence of overt respiratory disease, with reduced endothelium-dependent vasodilation independently associated with lower forced expiratory volume, supporting a shared vascular mechanism underlying cardiopulmonary impairment. Pulmonary vascular dysfunction reduces capillary perfusion and exacerbates ventilation–perfusion mismatch, impairing oxygen delivery and increasing susceptibility to hypoxemia, pulmonary edema, and postoperative respiratory failure [19].

Collectively, endothelial dysfunction and immune dysregulation in obese gynecological cancer patients promote myocardial oxygen supply–demand imbalance, vascular fluid leakage, and inflammatory lung injury, thereby increasing the risk of cardiopulmonary complications during the perioperative period and throughout chemotherapy and radiotherapy.

### 4.2. Metabolic Dysregulation and Cardiovascular Risk

Obesity is strongly associated with insulin resistance, dyslipidemia, and hypertension, three interrelated cardiometabolic abnormalities that synergistically drive adverse cardiovascular remodeling. Insulin resistance promotes compensatory hyperinsulinemia, which activates the sympathetic nervous system, enhances sodium retention, and impairs endothelial nitric oxide signaling, thereby contributing to vascular stiffness and hypertension [20]. Dyslipidemia, characterized by elevated triglycerides, increased low-density lipoprotein cholesterol, and reduced high-density lipoprotein cholesterol, accelerates atherosclerosis and disrupts coronary and systemic microvascular function, further compromising myocardial perfusion [21]. Concurrently, chronic hypertension increases left ventricular afterload, leading to concentric hypertrophy, myocardial fibrosis, and reduced ventricular compliance. In parallel, obesity is associated with expanded circulating blood volume and increased cardiac output to meet heightened metabolic demands. Persistent volume and pressure overload result in left ventricular hypertrophy and diastolic dysfunction, which impair ventricular filling and elevate intracardiac pressures. These structural and functional changes predispose obese patients to heart failure with preserved ejection fraction and atrial fibrillation [22]. Atrial remodeling and electrical instability further increase the risk of thromboembolic events and hemodynamic compromise [23].

In together, insulin resistance, dyslipidemia, and hypertension substantially reduce cardiac reserve and limit the heart’s ability to adapt to acute physiological stress. During the perioperative period and throughout chemotherapy and radiotherapy, additional stressors—including anemia, fluid shifts, and systemic inflammation—may precipitate myocardial oxygen supply–demand imbalance, arrhythmias, and acute heart failure. Consequently, obese gynecological cancer patients are particularly vulnerable to perioperative and treatment-related cardiovascular complications.

### 4.3. Obesity-Related Pulmonary Physiology

Obese gynecological cancer patients face respiratory challenges attributable to obesity-related alterations in lung mechanics, gas exchange, and respiratory muscle function. Excess adipose tissue within the thoracic and abdominal compartments significantly alters the mechanical properties of the lungs and chest wall, resulting in reduced pulmonary and chest wall compliance and increased overall respiratory system stiffness [24]. Increased respiratory stiffness also modifies normal breathing mechanics by limiting diaphragmatic descent and chest wall expansion, leading to mildly elevated intra-abdominal and pleural pressures compared with non-obese individuals [25]. As a consequence, expiratory reserve volume and functional residual capacity are markedly reduced [26]. Reduced functional residual capacity disrupts normal ventilation distribution, producing ventilation–perfusion mismatch and impaired arterial oxygenation, with preferential ventilation of nondependent lung regions and relative hypoventilation of well-perfused dependent zones [27].

Obstructive sleep apnea (OSA) represents an additional and highly prevalent contributor to pulmonary dysfunction in obese gynecological cancer patients. It is characterized by repetitive upper airway collapse during sleep, resulting in recurrent episodes of nocturnal hypoxia, hypercapnia, and heightened sympathetic activation, all of which may compromise cardiopulmonary reserve. A prospective observational study using perioperative sleep oximetry demonstrated that approximately 50% of gynecologic oncology patients had undiagnosed OSA, with obesity, advanced age, and increased neck circumference identified as significant risk factors leading to an increased risk of respiratory complications such as desaturation, atelectasis, and pneumonia [28].

In summary, these pathophysiological alterations markedly increase the risk of hypoxemia not only during the intraoperative and postoperative periods—particularly in the supine position required for surgery—but also in non-surgical gynecological cancer patients, where conditions such as ascites and pleural effusions, commonly seen in advanced ovarian and metastatic disease, further exacerbate respiratory compromise.

### 4.4. Hypercoagulability and Cancer-Associated Thrombosis

The combined prothrombotic effects of obesity and cancer markedly increase the risk of perioperative and treatment-associated venous thromboembolism in gynecological cancer patients, representing another source of cardiopulmonary morbidity and mortality. Obesity independently predisposes patients to thrombosis through enhanced coagulation activity and impaired fibrinolysis. Elevated circulating levels of fibrinogen, factor VII, and plasminogen activator inhibitor-1 promote clot formation while simultaneously inhibiting clot breakdown, predisposing patients to deep vein thrombosis and pulmonary embolism [29]. Chronic inflammation and oxidative stress in obese patients further exacerbate this risk by inducing endothelial activation and platelet aggregation, effects that are particularly pronounced during periods of immobility such as during hospitalization or during the perioperative period [30].

In gynecological cancer patients, this obesity-related prothrombotic state is compounded by cancer-associated thrombosis. In particular, tumor cells promote systemic hypercoagulability through direct activation of the coagulation cascade, primarily via overexpression of tissue factors and cancer procoagulants, as well as through the release of tissue-factor-bearing microparticles that amplify thrombin generation. Cancer-associated thrombosis can be conceptualized as two overlapping processes: Type I hypercoagulability, characterized by impairment of endogenous anticoagulant pathways, and Type II hypercoagulability, driven by classical mechanisms including endothelial injury, venous stasis, and procoagulant activation [31].

## 5. Chemotherapy-Related Cardiovascular and Pulmonary Toxicity

Table 1 summarizes the bidirectional interplay between obesity-related pathophysiological mechanisms and gynecologic cancer treatments, illustrating how metabolic dysregulation, chronic inflammation, and endothelial dysfunction predispose patients to cardiopulmonary complications during surgery, chemotherapy, and radiotherapy. All chemotherapeutic and targeted agents discussed in this section—including platinum compounds, taxanes, anthracyclines, antiangiogenic agents, and selected targeted therapies—are approved and routinely used in the management of ovarian cancer across frontline, recurrent, and maintenance treatment settings.

Platinum-based chemotherapeutic agents are widely used in gynecological oncology and especially as a systemic therapy for epithelial ovarian, primary peritoneal, cervical, and advanced or recurrent endometrial cancer. Although these therapies have substantially improved survival, their use is increasingly linked to cardiovascular complications that contribute to morbidity and may adversely affect long-term survivorship. Table 1 summarizes the cardiovascular and pulmonary complications of chemotherapy agents in obese gynecological cancer patients. Among platinum agents, cisplatin carries a higher cardiovascular risk, with reported complications including myocardial ischemia, acute coronary syndromes [32], arrhythmias, conduction abnormalities, hypertension, thromboembolic events, and left ventricular dysfunction. These effects are mediated by endothelial injury, oxidative stress, direct myocardial toxicity, prothrombotic mechanisms, and electrolyte disturbances, particularly hypomagnesemia and hypokalemia [33], and may occur acutely or present as delayed cardiovascular disease months to years after treatment. Notably, a large cohort study reported a 11% incidence of venous thromboembolism within 12 months of initiating cisplatin-based chemotherapy in women with ovarian cancer (*N* = 1880) [34].

Carboplatin is generally considered less cardiotoxic and is therefore preferred in patients with ovarian or advanced endometrial cancers. Nonetheless, cardiovascular events, including hypertension, heart failure, and arrhythmias, have been described, especially in patients with pre-existing cardiovascular disease, cumulative platinum exposure, or concomitant cardiotoxic therapies. Repeated carboplatin administration, common in recurrent ovarian cancer, may also lead to subclinical myocardial dysfunction, often detectable only with advanced cardiac monitoring [33]. In addition, platinum-based chemotherapy has been associated with an increased risk of arterial events, such as coronary vasospasm and myocardial infarction, occurring during treatment or in the post-therapy period [22].

The clinical selection between cisplatin and carboplatin is particularly relevant in obese patients with gynecologic malignancies due to important differences in systemic and cardiopulmonary toxicity. Cisplatin is associated with higher rates of endothelial injury, electrolyte disturbances, nephrotoxicity, arterial and venous thromboembolic events, and acute coronary syndromes, which may be amplified in patients with obesity-related cardiometabolic disease. In contrast, carboplatin demonstrates a more favorable tolerability profile, with reduced cardiovascular and renal toxicity, and is therefore commonly preferred in patients with significant comorbidity burden or limited cardiopulmonary reserve [35].

In endometrial cancer, carboplatin-based regimens have largely replaced cisplatin in contemporary practice, particularly in patients with obesity, advanced age, or pre-existing cardiovascular disease, reflecting comparable oncologic efficacy and improved safety. In ovarian cancer, carboplatin remains the backbone of first-line and recurrent treatment, while cisplatin is used selectively in specific clinical scenarios [36]. Although direct comparative data stratified by obesity are limited in ovarian cancer, the higher incidence of cisplatin-associated cardiovascular and thromboembolic complications supports preferential use of carboplatin in obese and high-risk patients [37].

Secondly, taxanes, including paclitaxel and docetaxel, are frequently used in the systemic treatment of gynecological malignancies and exert their antineoplastic effects by disrupting microtubule function and inhibiting cell division. The most commonly reported cardiovascular toxicity associated with taxanes is cardiac arrhythmias, including QT interval prolongation, followed by bradycardia and atrial fibrillation [38]. Paclitaxel is particularly associated with asymptomatic sinus bradycardia, reported in up to 29% of patients, which is typically transient and self-limiting. However, clinically significant cardiotoxic events, including atrioventricular conduction disturbances, ventricular tachycardia, and myocardial ischemia, have been observed in approximately 5% of treated patients [38]. Mechanistically, paclitaxel reduces calcium amplitude and myocardial contractility in cardiac myocytes, contributing to bradycardia.

Doxorubicin is primarily used in the management of advanced or recurrent endometrial cancer and uterine sarcomas. According to the American Society of Echocardiography and the European Association of Cardiovascular Imaging, anthracycline-related cardiotoxicity most commonly presents as a decline in left ventricular ejection fraction (LVEF) >10% from the baseline and may progress to overt heart failure. Large cohort studies have demonstrated that approximately 9% of patients treated with anthracyclines develop cardiotoxicity over a mean follow-up period of five years, with nearly 98% of cases occurring within the first year after treatment [39]. The risk of cardiotoxicity is strongly dose-dependent, with a substantial increase in heart failure incidence when cumulative doxorubicin doses exceed 400–700 mg/m^2^ [40]. However, subclinical myocardial dysfunction has been reported at considerably lower cumulative doses. Importantly, patients with gynecological cancers often have pre-existing cardiovascular risk factors, including obesity, hypertension, diabetes mellitus, and metabolic syndrome, which further heighten their susceptibility to anthracycline-induced cardiotoxicity [41].

Targeted and hormonal therapies further contribute to cardiovascular risk. Anti-HER2 therapies, including monoclonal antibodies and tyrosine kinase inhibitors (TKIs), are increasingly relevant in subsets of endometrial and ovarian cancers with HER2 overexpression. Cardiovascular toxicity associated with these agents most commonly manifests as left ventricular systolic dysfunction and heart failure, typically without overt cardiomyocyte death. The underlying mechanism involves inhibition of neuregulin-1/ERBB signaling, a pathway essential for cardiomyocyte survival and contractile function. Clinically, these effects are often reversible, but the risk is significantly amplified when anti-HER2 therapies are administered concurrently or sequentially with anthracyclines, leading to higher rates of heart failure [42].

Antiangiogenic agents, including bevacizumab, that inhibit vascular endothelial growth factor (VEGF) are frequently used in the treatment of cervical cancer, ovarian cancer, and uterine sarcomas. These therapies have been associated with cardiovascular adverse effects, including hypertension, heart failure, and arterial thromboembolic events, with evidence suggesting a dose-dependent increase in risk [43].

In addition to their well-established cardiovascular effects, systemic therapies used in gynecologic oncology are also associated with clinically significant pulmonary complications, which further contribute to treatment-related morbidity and diagnostic complexity. Cardiopulmonary toxicities frequently coexist, share overlapping pathophysiologic mechanisms, and may present with nonspecific symptoms such as dyspnea, hypoxia, or chest pain, often complicating differentiation from infection, thromboembolic disease, or cancer progression.

Platinum-based chemotherapeutic agents rarely cause primary pulmonary toxicity. When respiratory complications occur, they are most commonly related to acute hypersensitivity reactions, particularly after repeated exposure. These reactions may manifest as bronchospasm, dyspnea, or hypoxemia during or shortly after infusion and are thought to result from immune-mediated mechanisms [44]. In contrast, taxanes are more commonly associated with intrinsic pulmonary toxicity. Taxane-induced interstitial pneumonitis generally develops in a subacute fashion, days to weeks following treatment initiation, and is characterized by progressive dyspnea, nonproductive cough, and occasionally fever. Radiographic findings frequently demonstrate bilateral ground-glass opacities or patterns consistent with organizing pneumonia [45]. Bevacizumab, a monoclonal antibody targeting vascular endothelial growth factor, is associated with distinct pulmonary risks related to its anti-angiogenic mechanism. It carries distinct pulmonary risks related to its anti-angiogenic effects on vascular integrity. Although uncommon, pulmonary hemorrhage and hemoptysis can be life-threatening and necessitate immediate discontinuation of therapy [46]. Additionally, bevacizumab is associated with an increased risk of venous thromboembolic events, and pulmonary embolism should be strongly considered in patients presenting with acute dyspnea or hypoxemia during treatment [47].

Overall, cardiopulmonary toxicity associated with systemic therapy in gynecologic oncology are heterogeneous, potentially overlapping, and occasionally life-threatening. Early recognition, multidisciplinary assessment, and coordinated management strategies are essential to optimize patient outcomes and minimize long-term sequelae in this growing population of cancer survivors.

## 6. Surgery-Related Cardiopulmonary Complications in Obese Gynecological Cancer Patients

Surgery remains the cornerstone of treatment for most gynecologic malignancies; however, in obese patients it is associated with a substantially increased risk of perioperative cardiopulmonary complications. Obese gynecologic cancer patients are particularly vulnerable due to the combined effects of obesity-related cardiovascular remodeling, systemic inflammation, and reduced cardiopulmonary reserve. Table 2 presents the approximate reported risk range of major cardiopulmonary complications and intensive care unit admission in gynecologic cancer patients according to body mass index and treatment modality, highlighting the substantially increased perioperative and treatment-related vulnerability of obese patients. While ovarian cancer surgery is often associated with the greatest surgical burden, obese patients undergoing surgery for endometrial and cervical cancers also face substantial cardiopulmonary risk. In endometrial cancer, high rates of obesity, metabolic syndrome, and diastolic dysfunction increase vulnerability to perioperative myocardial injury and postoperative pulmonary complications. Cervical cancer patients undergoing radical hysterectomy or pelvic exenteration may similarly experience increased cardiopulmonary stress, particularly when combined with prior radiation exposure or compromised baseline pulmonary function.

Major abdominal surgery imposes significant cardiovascular stress; the results of a prospective study including 24,000 patients undergoing major abdominal surgery indicated that nearly 25% of deaths within the first 30 postoperative days were attributable to cardiovascular complications [48]. Perioperative myocardial injury—often asymptomatic and identifiable only through transient elevations in cardiac troponin—is a well-recognized predictor of mortality after non-cardiac surgery and may develop in the absence of overt ischemic symptoms [49]. Importantly, even subclinical troponin elevation has been associated with poorer survival outcomes [50]. Obese patients, who frequently present with hypertension, diastolic dysfunction, and unrecognized coronary artery disease, are particularly susceptible to perioperative myocardial oxygen supply–demand imbalance and, consequently, to the development of cardiovascular complications during the perioperative period.

**Table 2:** Cardiopulmonary complications and intensive care unit admission risk according to body mass index and treatment modality. Reported values represent approximate ranges derived from representative observational studies and large cohort analyses rather than pooled estimates. These ranges are provided for descriptive purposes to illustrate the relative increase in cardiopulmonary risk associated with obesity across treatment modalities.

Complications and Risk of Admission in ICU	Baseline Risk (Normal BMI)	Obese Patients (BMI > 30)	Relative Risk Increase	References
**Risk following surgery**				
**Venous thromboembolism (VTE)**	2–3%	8–12% (up to 20–25% in ovarian cancer)	~3–4×	[51,52,53]
**Heart failure/myocardial injury**	1–2%	5–8%	~2.5–4×	[45,46,47]
**Postoperative pulmonary complications** (atelectasis, pneumonia, respiratory failure)	5–8%	15–25%	~2–3×	[24,26,50]
**ICU admission**	3–5%	10–15%	~2–3×	[45,50]
**Risk following chemotherapy (platinum based, taxane based)**				
**Venous thromboembolism (VTE)**	3–5%	10–15% (≈11% within 12 months with cisplatin in ovarian cancer)	~2–4×	[34,44]
**Heart failure/cardiotoxicity**	1–2%	4–7%	~2–3.5×	[33,37,38]
**Arrhythmias/ischemic events**	2–4%	6–10%	~2–3×	[35,36]
**Pulmonary toxicity** (pneumonitis, PE-related dyspnea)	1–3%	5–8%	~2–3×	[42,44]
**Risk following radiotherapy**				
**Venous thromboembolism (VTE )**	1–2%	3–6%	~2–3×	[7,44]
**Cardiac events** (ischemia, HF exacerbation, late vascular disease)	1–2%	3–5%	~2–2.5×	[7,40]
**Pulmonary complications** (radiation pneumonitis, reduced pulmonary reserve)	2–4%	6–10%	~2–3×	[7,42]
**Treatment interruptions due to toxicity**	5–10%	15–20%	~2×	[7]

Fluid management during major gynecologic oncologic surgery represents a critical determinant of cardiopulmonary outcomes. Extensive procedures, particularly cytoreductive surgeries, are often associated with substantial perioperative fluid administration. Evidence suggests that a positive fluid balance exceeding 3000 mL is linked to a significantly higher risk of major postoperative complications, including pulmonary edema [51], whereas goal-directed fluid strategy in agreement with Enhanced Recovery After Surgery (ERAS) pathways limits the possibility of major cardiopulmonary complications in perioperative period [52]. Gynecologic cancer patients with an increased body mass index frequently exhibit sarcopenic obesity, hypoalbuminemia, and pre-existing cardiac disease, and may also experience substantial intraoperative blood loss. Together, these factors diminish physiological reserve and impair effective fluid management, making this group of patients particularly vulnerable to perioperative fluid overload and increasing the risk of postoperative respiratory complications and cardiac dysfunction.

Pulmonary complications are among the most frequent adverse events in the perioperative period after a major gynecological cancer surgery. In a large prospective study of 165,196 patients undergoing major abdominal surgery, 5.8% developed postoperative pulmonary complications, including pneumonia, prolonged mechanical ventilation, or unplanned reintubation. Poor performance status, prolonged operative duration, ascites, smoking, advanced age (>80 years), and pre-existing pulmonary disease were identified as the strongest predictors of postoperative pulmonary morbidity [53]. In obese patients, these risks are further amplified by obesity-related reductions in functional residual capacity, impaired diaphragmatic excursion, and ventilation–perfusion mismatch, which, when combined with anesthesia and prolonged operative time, substantially increase the likelihood of hypoxemia, atelectasis, and postoperative respiratory failure.

Another major adverse outcome during the perioperative period in gynecological cancer patients is venous thromboembolism (VTE), with obesity serving as a key predisposing factor. In ovarian cancer, the incidence of deep vein thrombosis has been reported to reach approximately 25% [54], while rates in endometrial cancer range from 0.8% to 8.1% [55] and in cervical cancer from 0.7% to 12.3%. Advanced age, obesity, reduced performance status, and postoperative complications further increase thromboembolic risk. In a prospective study of 7266 patients undergoing minimally invasive gynecological surgery for gynecological malignancy, the risk of VTE was four times higher in the group of obese patients compared to the normal-weight group, and for each 5 kg/m^2^ increase in BMI, the risk of VTE increased by 21.5% (RR 1.215, 95% CI 1.09–1.36) [56]. Importantly, thromboprophylaxis strategies in morbidly obese patients remain challenging, as standard fixed-dose regimens may lead to inadequate anticoagulant exposure. Many institutions adopt weight-adjusted prophylactic dosing approaches, particularly for low-molecular-weight heparin, to reduce underdosing risk in patients with extreme BMI [57]. In parallel, direct oral anticoagulants (DOACs) are increasingly used in cancer-associated thrombosis; however, their role in morbid obesity remains an evolving area due to concerns regarding pharmacokinetic variability and limited high-quality evidence in this subgroup [58,59].

Overall, obese gynecologic cancer patients undergoing surgery constitute a high-risk population for cardiopulmonary complications. Table 3 summarizes the estimated cardiopulmonary risk profiles of obese patients undergoing multimodal treatment for endometrial, ovarian, and cervical cancers, demonstrating substantial variation in thromboembolic, cardiac, and pulmonary complications across gynecologic malignancies. The convergence of obesity-related cardiometabolic disease, cancer-associated hypercoagulability, and the physiological stress of major surgery underscores the importance of meticulous perioperative assessment based on ERAS protocols, goal-directed fluid management, and appropriate use of high-dependency or intensive care monitoring to mitigate adverse outcomes.

## 7. Future Directions and Conclusions

The growing population of obese gynecological cancer patients underscores the urgent need for structured strategies to mitigate cardiopulmonary morbidity and mortality. Future directions should prioritize applying risk stratification models that integrate obesity-related metrics (BMI, body composition, sarcopenic obesity), cardiovascular comorbidities, functional capacity, and cancer-specific factors to identify patients at highest perioperative and long-term risk. Incorporation of biomarkers of myocardial injury, cardiopulmonary reserve testing, and standardized preoperative cardiovascular and pulmonary assessment may further enhance individualized risk stratification.

In clinical practice, perioperative and long-term risk stratification may be supported by established cardiovascular and surgical risk assessment tools, such as the Revised Cardiac Risk Index, perioperative myocardial injury surveillance strategies, and functional capacity assessments [60]. Although not specifically developed for oncologic or obese populations, these tools may provide a structured framework for identifying patients who may benefit from intensified preoperative optimization, cardiology consultation, and enhanced postoperative monitoring.

Preoperative optimization represents a critical opportunity to improve outcomes. Targeted management of obesity-related conditions, including hypertension, diabetes, obstructive sleep apnea, and heart failure, should be systematically adopted prior to surgery and systemic therapy. Multidisciplinary prehabilitation programs, incorporating nutritional optimization, weight management, physical conditioning, and smoking cessation, may improve cardiopulmonary reserve and reduce postoperative complications. In high-risk patients, early involvement of cardiology, pulmonology, and anesthesia teams is essential to guide tailored perioperative planning. In addition to thrombotic risk, global patient frailty and bleeding risk warrant consideration in obese gynecologic cancer patients. Advanced age, anemia, renal dysfunction, nutritional impairment, and prior bleeding events frequently coexist with obesity-related cardiometabolic disease and may further increase vulnerability to perioperative complications, anticoagulation-related bleeding, and treatment intolerance. Integrating bleeding risk within a broader frailty framework may support more individualized perioperative and long-term management strategies in this population. Bleeding risk assessment tools developed in cardiovascular medicine, such as the PRECISE-HBR score, incorporate variables closely related to frailty and comorbidity, including age, renal function, anemia, and prior bleeding, and may offer conceptual insight when evaluating overall clinical vulnerability in obese gynecologic cancer patients [61].

In addition to lifestyle interventions and bariatric surgery, pharmacologic obesity management is increasingly relevant in gynecologic oncology, particularly with the growing use of glucagon-like peptide-1 receptor agonists (GLP-1 RAs). These agents may improve cardiometabolic risk profiles through weight reduction and improved glycemic control, potentially offering indirect cardiopulmonary protection in high-risk obese patients. However, perioperative management requires caution, as GLP-1 RAs may delay gastric emptying and could increase the risk of perioperative regurgitation and aspiration during anesthesia. Thus, coordinated perioperative planning between oncology, anesthesia, and metabolic specialists is recommended when these therapies are used in surgical candidates.

In parallel, implementation of Enhanced Recovery After Surgery (ERAS) protocols, with particular attention to goal-directed fluid therapy, opioid-sparing analgesia, early mobilization, and optimized respiratory support, offers considerable potential to reduce cardiopulmonary stress in obese patients undergoing gynecologic cancer surgery. From a perioperative perspective, selective admission to high-dependency or intensive care units should be based on structured preoperative risk stratification rather than reactive management following clinical deterioration. Early postoperative monitoring of high-risk patients may facilitate prompt detection of myocardial injury, respiratory compromise, and thromboembolic events, thereby reducing preventable morbidity.

In conclusion, obesity significantly amplifies cardiopulmonary risk in gynecological cancer patients through a complex interplay of metabolic dysfunction, cancer biology, treatment-related toxicity, and surgical stress. Addressing this challenge requires an integrated, multidisciplinary approach centered on prevention, early identification of high-risk patients, and evidence-based perioperative strategies. Future research should focus on the validation of risk stratification tools, the optimization of performance status and management of comorbidities in this patient population, and the development of effective perioperative and long-term cardiopulmonary care models to improve both oncologic and non-oncologic outcomes in this vulnerable group.

## Figures and Tables

**Table 1:** Interplay between obesity, gynecologic cancer treatment, and cardiopulmonary complications. Obesity promotes metabolic dysregulation, chronic low-grade inflammation, endothelial dysfunction, and a prothrombotic state, leading to reduced cardiac and pulmonary reserve in patients with gynecologic malignancies. These obesity-related alterations increase susceptibility to cardiopulmonary complications during surgery, chemotherapy, and radiotherapy. In turn, cancer treatments further exacerbate cardiac and pulmonary injury through inflammatory, hemodynamic, and direct toxic mechanisms, creating a bidirectional cycle that amplifies peri-treatment morbidity, treatment intolerance, and adverse clinical outcomes, including increased cardiopulmonary morbidity, treatment delays, and reduced survival and quality of life. ↑, increase.

Component	Obesity-Related Mechanisms	Interaction with Cancer Treatment	Resulting Cardiopulmonary Complications	Clinical Impact
**Obesity**	Visceral adiposity/Insulin resistance & hyperinsulinemia/Dyslipidemia/Chronic low-grade inflammation (↑ IL-6, TNF-α, CRP)/Endothelial dysfunction/Prothrombotic state	Primes patients for increased vulnerability to surgical stress, cardiotoxic therapies, and radiation-induced vascular injury	Reduced cardiac and pulmonary reserve/Increased myocardial oxygen demand/Impaired gas exchange	Establishes high baseline cardiopulmonary risk prior to treatment
**Surgery**	Hypertension, LV hypertrophy, diastolic dysfunction/Reduced lung compliance & FRC/Obstructive sleep apnea/Hypercoagulability	Major surgical stress/Inflammatory surge/Fluid shifts and volume overload/Prolonged anesthesia & immobility	Perioperative myocardial injury/Acute heart failure & arrhythmias/Pulmonary edema, atelectasis, pneumonia/Venous thromboembolism/pulmonary embolism	↑ ICU admission/↑ Length of stay/↑ Postoperative morbidity & mortality
**Chemotherapy**	Pre-existing cardiometabolic disease/Endothelial dysfunction/Reduced cardiopulmonary reserve	Direct myocardial toxicity (platinum agents, anthracyclines)/Endothelial injury/Fluid retention/Prothrombotic effects	Heart failure (often HFpEF)/Arrhythmias/Ischemic events/Pulmonary toxicity (pneumonitis, PE)	Treatment dose reductions or delays/Reduced treatment tolerance/Increased non-cancer mortality
**Radiotherapy**	Chronic inflammation/Vascular dysfunction/Impaired tissue repair capacity	Radiation-induced endothelial injury/Accelerated atherosclerosis/Pulmonary inflammatory injury	Radiation-associated cardiotoxicity/Pulmonary fibrosis/Worsening dyspnea and exercise intolerance	Treatment interruptions/Increased late cardiopulmonary toxicity
**Bidirectional amplification**	Obesity-related inflammation and metabolic dysfunction persist throughout treatment	Surgery, chemotherapy, and radiotherapy further exacerbate cardiopulmonary injury	Progressive cardiac and pulmonary dysfunction	Vicious cycle leading to cumulative morbidity

**Table 3:** Estimated cardiopulmonary risk profiles in obese patients undergoing multimodal treatment for gynecologic malignancies. Risk estimates reflect literature-informed ranges based on published cohort studies and are intended to demonstrate relative differences in cardiopulmonary burden across cancer types rather than precise pooled risks.

Cancer Type	Typical Multimodal Treatment	VTE Risk (%)	Heart Failure/Myocardial Injury Risk (%)	Pulmonary Complication Risk (%)	Overall Cardiopulmonary Risk Profile	References
**Endometrial cancer**	Surgery ± adjuvant radiotherapy ± chemotherapy (selected cases)	6–10%	5–8% (HFpEF, perioperative myocardial injury)	10–15% (atelectasis, pneumonia, hypoxemia)	**High** Cardiovascular risk often exceeds cancer mortality long-term	[4,12,45,52]
**Ovarian cancer**	Extensive cytoreductive surgery + platinum-based chemotherapy ± maintenance therapy	10–25%	6–10% (chemo-related cardiotoxicity, myocardial injury)	15–30% (pleural effusion, PE, respiratory failure)	**Very High** The most severe cumulative cardiopulmonary burden is present in this group	[3,34,45,51]
**Cervical cancer**	Surgery (early stage) or chemoradiation (locally advanced)	4–8%	3–6% (radiation-related vascular injury, HF exacerbation)	8–15% (radiation pneumonitis, PE, dyspnea)	**Moderate****–****High** Risk driven by combined chemoradiation	[7,40,44]

## Data Availability

No new data were created or analyzed in this study.
